# Pre-Post Evaluation of a Sun Safety Social Media Campaign for Young Adults to Reduce Skin Cancer Risk

**DOI:** 10.2196/90595

**Published:** 2026-07-07

**Authors:** Janessa M Mendoza, David Perez, Muriel R Statman, Mia K Price, Madeleine F Brown, Omar U Anwar, Marcelo M Sleiman Jr, Kenneth P Tercyak

**Affiliations:** 1 Lombardi Comprehensive Cancer Center, Georgetown University, 2115 Wisconsin Avenue, NW, Washington, DC, 20007, United States, 1 202-687-0802; 2 School of Medicine, Georgetown University, Washington, DC, United States

**Keywords:** sun safety, social media, young adults, skin cancer prevention, health communication, digital health promotion

## Abstract

**Background:**

Social media is a prevalent source of health information for young adults and offers a scalable platform for skin cancer prevention messaging, including sun safety. Despite high awareness among young adults that UV radiation is a potent carcinogen, both intentional and unintentional exposure remain common. Group-targeted and tailored digital campaigns may help counter protanning norms and strengthen protective behaviors to reduce long-term risk.

**Objective:**

This study aimed to evaluate the reach, engagement, and influence of a group-targeted and tailored Instagram-based sun safety campaign implemented as part of a university Skin Smart Campus (SSC) initiative.

**Methods:**

A pre-post design was used during the 2024‐2025 academic year. The social media campaign evaluation was a focused analysis within a broader sun safety implementation initiative reported elsewhere. The analysis covered the trend-aligned Instagram campaign’s reach, engagement, awareness, aided thematic recall, following, and associations with sun safety behaviors. Surveys assessed demographics, sun safety behaviors, campaign awareness, and content recall. Instagram analytics quantified reach and engagement, and influence analyses included bivariate tests and logistic regression.

**Results:**

Presurveys and postsurveys were obtained anonymously from 230 and 267 participants, respectively. A dedicated Instagram account and n=10 campus organizations shared campaign content, extending potential reach to 5711 campus followers. Weekly educational posts averaged 262 views and reached approximately 38 nonfollowers per post throughout the campaign. Postcampaign, SSC social media awareness was reported by 48.7% (130/267) of participants, compared to 24.1% (55/230) precampaign (*χ²*_1_=32.5; *P*<.001). There was significantly higher awareness among those with lighter skin types (93/164, 56.7%) compared to darker skin types (37/103, 35.9%; *χ²*_1_=10.9; *P*<.001). Followers were more likely to use on-campus sunscreen dispensers (*χ²*_1_=85.2; *P<*.001). Sunscreen use, protective clothing, and tanning bed risks were among the most frequently posted and recalled themes on Instagram, suggesting high salience (121/124, 97.6%; 111/124, 89.5%; and 106/124, 85.5%, respectively). Multivariable logistic regression revealed that participants with lighter skin tones (OR 2.33, 95% CI 1.19‐4.56; *P*=.01), who followed the SSC Instagram account (OR 6.81, 95% CI 3.41‐13.60; *P*<.001), and who used sunscreen dispensers (OR 7.78, 95% CI 3.82‐15.84; *P*<.001) were significantly more likely to practice greater sun safety.

**Conclusions:**

A group-targeted, tailored social media campaign embedded in a broader campus initiative demonstrated meaningful reach, engagement, message recall, and impact among young adults. Pairing digital strategies with environmental ultraviolet radiation-reducing resources may enhance young adults’ sun safety behaviors. Future efforts should address differences in social media campaign awareness and strengthen messaging for groups that perceive themselves to be at lower risk for skin cancer.

## Introduction

Social media has rapidly become one of the most influential channels for health communication, offering opportunities to disseminate information with speed, personalization, and interactive engagement that surpass traditional media. Its scale, peer-to-peer connectivity, and algorithm-driven reach make it a particularly powerful platform for health behavior impact [1].

Young adults consistently rely on social media for health information because it is convenient, relatable, and easier to access than formal medical sources [1]. While data from 2022 indicated that over two-thirds of young adults aged 18 to 24 years used social media to learn about their health [2], this proportion has likely increased since then, as digital channels continue to outpace traditional media in speed, personalization, and accessibility. For example, Stifjell et al [3] reported that social media use among young adults outpaced search engine use when seeking health information. Video-based platforms, such as YouTube, Instagram, and TikTok, are especially appealing, as their dynamic and narrative formats facilitate understanding and enhance engagement compared with written materials [2]. Importantly, health information is encountered both actively and passively: many young adults are exposed to algorithm-driven health-related posts by chance on newsfeeds or advertisements, while others join groups or follow pages for specific health topics [2]. Beyond information-seeking, young adults also use social media for peer support and community-building, making digital platforms appear more relatable and accessible than formal medical or public health sources [4]. Thus, digital platforms act as potent tools in shaping young adults’ norms, attitudes, and behaviors regarding health-related topics and public health concerns [4].

One such concern is skin cancer prevention, as skin cancer remains the most frequently diagnosed cancer worldwide, despite its largely preventable nature. Approximately 6.1 million individuals are treated annually with an estimated US $8.9 billion in associated annual health care costs in the United States alone [5]. Melanoma, the deadliest form of skin cancer, is one of the most frequently diagnosed cancers among adults aged 20 to 29 years [6,7] and is more frequently diagnosed in women before the age of 50 years [8]. These statistics reinforce the critical importance of skin cancer prevention efforts among young adults.

Because UV radiation (UVR) from sunlight or indoor tanning is a carcinogen, it remains the primary modifiable cause of skin cancer risk [9-12], and targeted, tailored behavioral interventions are critical to reducing long-term exposure and preventing disease [13]. Young women often desire tan skin to improve their appearance or self-confidence, particularly in anticipation of significant social gatherings [14,15]. The ubiquity of this beauty ideal is evidenced by the fact that over 90% of female undergraduates believe they look better with tan skin, with 1 in 5 choosing to indoor tan before special events despite acknowledging the risk of skin cancer [16]. Social media further amplifies these risks by disseminating protanning messages and misinformation about tanning benefits [17-20], consequently fostering favorable attitudes toward tanning and increased tanning behavior among young adults [21,22]. Despite widespread awareness among young adults that excess UVR is a potent skin cancer-causing agent [16,23], 51% of young adults younger than the age of 25 years report annual sunburns, accompanied by inconsistent sunscreen use and reapplication [24]. This pattern is particularly alarming because the relationship between sunburns and skin cancer is strong: even a single sunburn doubles an individual’s lifetime risk of developing melanoma, resulting in substantial health care usage and high costs [5,25]. This persistent burden is exacerbated by a behavioral paradox where young adults with higher health literacy exhibit high incidental UVR exposure and low sunscreen adoption [23], highlighting the need for prevention approaches responsive to the real-world social environments where these behaviors occur.

Given these challenges, social media–based campaigns have emerged as promising strategies to counteract protanning messaging and promote sun safety behaviors among young adults. The eHealth behavior change framework emphasizes the importance of theory-driven, audience-tailored health communications [26]. Message tailoring (customizing content to individual attributes such as UVR risk awareness) has been shown to be particularly effective [27]. Combining tailored and targeted approaches, where messages are designed for specific subgroups like young adults, optimizes both resource efficiency and message impact [28]. Trend-based, visual, and memetic formats further enhance engagement and recall among young adults, leveraging perceived relevance, normative influence, and narrative persuasion [28-31].

Empirical evidence supports the effectiveness of social media health campaigns in changing behavior. For instance, Facebook-based interventions have increased condom use [32] and smoking cessation rates [33,34] among young adults. In the context of skin cancer prevention, digital interventions have led to increased sun protection, sunscreen use, and motivation to stop tanning indoors [35,36]. Social media has also served as a motivation for seeking dermatological care, with 31.7% of first-time patients undergoing dermoscopy reporting referral via social platforms [37]. In addition to influencing behavior, social media provides a feasible environment for evaluating the reach and impact of health interventions [38]. Metrics such as likes, views, shares, and impressions are commonly used to assess engagement and audience interaction [38-40]. These analytics offer valuable insights into the effectiveness of digital campaigns and may inform future strategies for public health messaging.

Taken together, evidence from prior research supports the potential of social media–based strategies in promoting sun safety and reducing skin cancer risk among young adults. The broader implementation outcomes of this university’s Skin Smart Campus (SSC) initiative, including pre-post changes in sun safety knowledge, sunscreen dispenser use, and overall sun safety behavior, have been reported previously [41]. Building on that implementation evaluation, the present manuscript focuses specifically on the social media component of the SSC initiative. This study evaluates the reach, engagement, awareness, aided thematic recall, and behavioral correlates of a group-tailored, targeted, and theory-driven social media campaign in a university-based young adult population, addressing both the promise and complexity of digital health promotion in this high-risk group. Specifically, this study examines how participants interacted with campaign content, which campaign themes were recalled, how Instagram awareness and following were associated with sun safety behaviors, and how social media metrics can be used to assess the reach and influence of prevention messaging.

The broader SSC initiative included campus designation, educational web materials, small media, sunscreen dispensers, signage, and Instagram-based communication; its implementation outcomes, including knowledge change, dispenser use, and pre-post sun safety behavior comparisons, are reported elsewhere [41]. The analysis focuses on social media–specific campaign indicators, including Instagram reach and engagement, SSC Instagram awareness, aided thematic recall, and associations between Instagram following and sun safety behaviors.

## Methods

### Study Design and Setting

This study used an anonymous, independent pre-post design to evaluate the impact of a multicomponent, longitudinal sun safety public health education campaign among young adults. The campaign was conducted at a private university during the 2024‐2025 academic year. The project was part of the institution’s designation as an SSC, a national initiative led by the National Council on Skin Cancer Prevention that recognizes schools for their commitment to reducing UVR exposure among young adults [41]. All SSC infrastructure, including some campus sunscreen dispensers, was in place prior to the baseline assessment period. Anonymous, independent presurveys and postsurveys were administered online such that participant overlap could not be determined. The surveys collected data on respondents’ sun safety knowledge, behaviors, and campaign awareness over time.

### Participants and Recruitment

Eligible participants were aged 18 years or older and enrolled at the university through an undergraduate, graduate, or professional degree program. Recruitment occurred through university-managed social media, tabling events, and campus organization listserv emails. This approach was selected for its feasibility and alignment with evaluating a campus-based public health campaign in a real-world university setting. All survey data were collected using a mobile-friendly online platform (Qualtrics). To support eligibility verification while maintaining anonymity, access to the survey required a university-affiliated email login, and eligibility criteria (age and enrollment status) were self-reported. The study protocol was reviewed and deemed exempt by the university’s institutional review board.

### Campaign Components

#### Digital Environment: Group-Targeted and Tailored Social Media Campaign

##### Overview

The SSC social media campaign was both group-targeted and tailored [42,43]. Group-targeting is designed for a defined subgroup whose members share a characteristic relevant to the behavior or risk context, such as age, race or ethnicity, occupation, or disease risk; the same core content is typically delivered to everyone in that subgroup [43]. Relatedly, group-tailoring goes beyond selecting a subgroup audience and adapts content, delivery, framing, examples, activities, or implementation strategies to the group’s assessed needs, shared identity, cultural context, barriers, resources, and mechanisms of change [42]. When used together, group targeting and group tailoring can be synergistic: targeting helps ensure that the campaign reaches a subgroup with shared needs, while tailoring increases its fit, relevance, and feasibility for that group’s real-world context [44]. Campaigns that both identify an appropriate priority group and adapt strategies to that group’s circumstances may have stronger effects than those that rely on targeting or tailoring alone, particularly when behavior change depends on social norms, environmental supports, and setting-specific barriers [42-44]. This distinction is important in skin cancer prevention because outdoor settings are a priority context for reducing UVR exposure, and multicomponent approaches that combine health education, behavioral supports, environmental changes, and policies have strong evidence for improving sun safety behaviors [45].

This study reports the social media–specific tailoring process at the level needed to interpret campaign content and engagement. Detailed implementation outcomes for the broader SSC initiative are reported elsewhere [41]. Briefly, baseline survey data identified communication channels used by students and highlighted sun safety knowledge and behavior gaps relevant to campaign planning, including misconceptions about UVR exposure from tanning beds, inconsistent sunscreen use and reapplication, and infrequent use of protective clothing and hats. These findings informed the selection of campaign themes and the emphasis placed on sunscreen use, sunscreen reapplication, tanning bed risks, sun-protective clothing, and UVR risk across skin tones.

##### Precampaign Planning and Platform Selection

Precampaign (baseline) data on participants’ preferred communication channels, as well as their sun safety knowledge and behaviors, guided the group-tailored development of the SSC social media campaign to align with participants’ social media usage patterns and educational needs. Instagram was selected because it is one of the most commonly used social media platforms in the United States and is particularly well-suited to short-form video content. Although YouTube is the most widely used platform among US adults, Instagram is considered especially behaviorally influential due to its highly visual interface and the ease with which users can continuously scroll through short-form content [46].

Baseline survey findings also informed the educational priorities of the campaign. For example, at baseline and as described in more detail by Perez et al [41], nearly one-fifth of respondents (18.9%) incorrectly believed that the sun emits more UVR than tanning beds. In addition, 15% reported never or rarely using sunscreen with SPF 30 or higher, 53.5% reported never or rarely reapplying sunscreen appropriately, 49.2% reported never or rarely wearing hats, and 34.2% reported never or rarely wearing clothing that covers the arms or legs. These findings informed campaign messaging emphasizing tanning bed risks, sunscreen use and reapplication, and sun-protective clothing [41]. Because these knowledge and behavior outcomes are reported previously, they are summarized here only to describe how baseline data informed the SSC Instagram campaign.

##### Campaign Implementation

Campaign content was thus designed to reflect participants’ daily routines, lived experiences, and common misconceptions about UVR exposure. Posts were intentionally aligned with current social media consumption patterns among young adults, incorporating concise captions and text overlays, humor-based and meme-style graphics, and short-form videos inspired by contemporary social media trends to enhance relatability, credibility, and algorithmic reach.

At the start of the fall semester and continuing through the spring semester, a total of 62 Instagram posts were shared, including 43 educational posts and 19 outreach posts. Outreach posts promoted SSC-related events, including tabling sessions designed to encourage survey participation and distribute sun safety materials (eg, educational handouts, UV bracelets, and sunscreen lip balm). Posts were distributed weekly, with increased frequency to twice weekly during relatively higher UVR months (August to October) and before and during the university’s spring break week in March.

##### Peer Network Dissemination Strategy

To expand the reach of the social media campaign beyond the SSC Instagram account and its followers, a structured peer network dissemination strategy was implemented. A total of 85 outreach emails were sent to individuals involved in 75 (n=34 campus clubs and n=41 athletics-affiliated groups) unique academic organizations, cultural clubs, athletic teams, performance groups, and affinity groups registered with the university. Because these organizations had established digital networks, they were invited to disseminate SSC Instagram content through their own platforms. Organizations that shared SSC Instagram content were tracked using Instagram’s “Story Reshares” and “Mentions” analytics, as well as manual logs of confirmed reposts.

##### Retrospective Content Coding

To systematically characterize the delivered campaign content, all SSC Instagram posts were retrospectively coded using a content matrix developed by the study team. Posts were individually reviewed and classified according to the dominant theme (eg, sunscreen use, sun-protective clothing, winter sun safety, skin cancer across skin tones, and indoor tanning risk) and format (post, 24-hour story, or reel). Posts could contain more than 1 theme; therefore, theme counts were not mutually exclusive. The coding approach was designed to support the present manuscript’s social media evaluation by linking delivered campaign themes to Instagram engagement metrics and postcampaign aided thematic recall. Creative executions and message-framing examples, such as humor, trend-aligned audio, or appearance-related depictions of sun damage, were considered during interpretation but were not treated as separate quantitative outcome categories. Two independent coders reviewed all content, and interrater reliability was assessed using percent agreement. Discrepancies were resolved through discussion and consensus with a third reviewer. This systematic coding approach enabled subsequent evaluation of participant content recall, which served as a dependent variable in the postcampaign survey.

### On-Campus Sunscreen Dispensers and Signage

Social media posts also promoted other components of the university’s SSC designation, including on-campus sunscreen dispensers, signage, and online educational materials. As part of the university’s SSC designation, skin cancer prevention information was included on the student health center’s website, and related studies were published in the student newspaper and a campus newsletter. These materials highlighted the four publicly accessible on-campus sunscreen dispensers installed in high-traffic indoor locations (eg, dining areas) to increase access to UVR protection. These installations occurred prior to the deployment of the presurvey and before the start of the social media campaign. Given the high levels of sun exposure associated with physical activity and exercise, 3 additional sunscreen dispensers were installed at athletic facilities during the campaign period, increasing the total number of dispensers to seven. Educational signage accompanied the dispensers and was displayed throughout campus, including residence halls and academic buildings, to reinforce daily sunscreen use and promote year-round sun safety. This information was also clearly shared on the SSC Instagram account. Additional implementation details, including dispenser placement, are described by Perez et al [41].

### Campaign Evaluation

#### Independent Variables

##### Demographics

In both presurveys and postsurveys, demographics were collected, including age, gender, race or ethnicity, and program affiliation (undergraduate, graduate, or professional).

##### Sunscreen Dispenser Use

Participants were asked whether they had ever used an on-campus sunscreen dispenser, with responses coded dichotomously (yes or no) in presurveys and postsurveys.

##### Sun Safety Behaviors

Sun safety behaviors were assessed in presurveys and postsurveys using a 7-item composite measure, rated on a 5-point Likert scale (0=“never” to 4=“always”), adapted from prior research [47]. The validation, reliability, and limitations of these measures within the SSC initiative were previously established and are described in detail elsewhere [41]. Cronbach α indicated acceptable internal consistency for the scale, with an overall value of 0.70 (0.57 at presurvey, 0.83 at postsurvey). Items included sunscreen use and its reapplication, protective clothing use, hat use, seeking shade, and checking the UV index. Higher summed scores indicated greater engagement in sun-protective behaviors.

##### Skin Type

In the postsurvey only, information was collected on young adults’ Fitzpatrick Scale skin type ranging from I to VI, based on a person’s self-reported tendency to sunburn and tan; higher values correspond to darker skin tones and a lesser tendency to sunburn [48,49].

##### Athletic Facility Usage

Given the locations of the new sunscreen dispensers at athletic facilities, participants reported the frequency of visiting campus athletic facilities, which was coded categorically on a 5-point Likert scale (0=“never” to 4=“always”) in the postsurvey.

### Dependent Variables

#### Instagram Metrics

Engagement with the SSC Instagram account was evaluated using metrics obtained from Instagram Insights, a built-in analytics tool available to users with a business or creator account. This tool provided data on how the posted content and audience were performing to optimize the SSC Instagram account’s reach (exposure) and impact (interaction). Analytics included total followers and nonfollowers who engaged with content, likes per-post, views or impressions (including repeat views), and shares of content. Based on prior literature on social media health promotion campaigns [38-40], likes and views were prioritized as primary engagement indicators, with shares or comments monitored but anticipated to be less frequent in this context. These metrics reflect platform-defined measures of reach and engagement that are generated through Instagram’s proprietary algorithms and reporting structures. Metrics are provided in aggregate form and summarize interactions across followers and nonfollowers. As such, these measures characterize overall content performance and audience interaction patterns at the account level rather than tracking exposure at the individual user level.

#### SSC and Instagram Awareness

In the precampaign survey, participants were asked about their awareness of the university’s SSC initiative. In contrast, the postcampaign survey distinguished between general awareness of the initiative and awareness of the public SSC Instagram specifically; the latter was assessed using 2 items: participants were first asked whether they were aware of the university’s SSC social media campaign on Instagram (yes or no) and, if so, whether they followed the public account (yes or no). SSC Instagram awareness served as the primary indicator of campaign reach. Because the SSC Instagram-specific following item was administered only postcampaign, baseline Instagram following could not be assessed.

#### Content Recall

In the postsurvey, participants who reported awareness of the SSC Instagram account were presented with a list of campaign content themes and were asked to select the themes they recalled seeing. This measure assessed aided thematic recall rather than unaided recall of specific posts, captions, or graphics. Aided recall was included as a social media–specific indicator of message salience and campaign penetration, particularly because Instagram analytics were available only in aggregate form and could not be linked to individual survey respondents.

### Data Analysis

Cross-sectional postsurvey data were analyzed using SPSS version 29.0. Descriptive statistics were used to summarize demographic and behavioral characteristics. Chi-square tests assessed categorical variables (eg, dispenser use by Instagram followers). Independent samples *t* tests compared sun safety behaviors across other factors (eg, skin tone, Instagram following, and dispenser use). Finally, logistic regression analysis was used to identify cofactors of sun safety. The model’s independent variables were selected based on their potential relevance to UVR risk perception, previous evidence that social media health campaigns impact cancer-preventive behaviors, and bivariate associations observed within the postsurvey dataset (*P*<.10) [50].

### Ethical Considerations

This study was exempt from review by the Georgetown University Institutional Review Board (STUDY00008246). Informed consent was obtained from all participants involved in the study. The study protocol was reviewed and deemed exempt by the university’s Institutional Review Board. All data were collected and stored in compliance with the Health Insurance Portability and Accountability Act (HIPAA).

## Results

### Peer Network Campaign Outreach

In fall 2024, the university enrolled approximately 7800 undergraduate and 8900 graduate or professional students, totaling an estimated 16,700 students. Of the 85 outreach emails sent to 75 unique university organizations, ultimately 10 with established Instagram accounts reposted SSC Instagram content. This extended the potential reach to 5711 Instagram followers, or roughly one-third of the student body.

### Participant Characteristics

A total of 230 participants completed the precampaign survey and 267 completed the postcampaign survey (Table 1). Due to the anonymous and independent nature of the surveys, some participant overlap may have occurred. As illustrated in Table 1, presurvey and postsurvey demographic characteristics were comparable [41]; they are presented to help characterize the samples and provide context for the social media–focused analyses.

**Table 1.** Participant demographic data.

Factor	Precampaign (n=230)^a^	Postcampaign (n=267)^a^
Age (y), mean (SD)	23.7 (3.1)	23.3 (3.0)
Sex, n (%)		
Female	160 (69.6)	176 (65.9)
Male	68 (29.6)	91 (34.1)
Race, n (%)		
Asian or Pacific Islander	76 (33.0)	80 (30.0)
Black or African American	21 (9.1)	17 (6.4)
Middle Eastern	22 (9.6)	22 (8.2)
Other	32 (13.9)	27 (10.1)
White	79 (34.3)	121 (45.3)
Ethnicity, n (%)		
Non-Hispanic	212 (92.2)	242 (90.6)
Hispanic	18 (7.8)	25 (9.4)
Skin type^b^, n (%)		
I (“Very fair”)	—^c^	70 (26.2)
II (“Fair”)	—	94 (35.2)
III (“Light-medium”)	—	60 (22.5)
IV (“Medium/olive”)	—	24 (9.0)
V (“Brown”)	—	15 (5.6)
VI (“Deep brown or black”)	—	4 (1.5)

a These samples are presumed to be independent, but cannot be entirely verified.

b Assessed at postcampaign only.

c Not applicable.

Precampaign participants had a mean age of 23.7 (SD 3.1), were predominantly female (160/230, 69.6%), and represented a diverse racial composition (Asian or Pacific Islander: 76/230, 33.0%; Black or African American: 21/230, 9.1%; Middle Eastern: 22/230, 9.6%; Hispanic: 18/230, 7.8%). More than half of the participants were enrolled in a health profession program (150/230, 65.2%). The majority reported keeping up with university news through emails (199/230, 86.5%) or university-managed social media accounts (107/230, 46.5%), highlighting the prominence of digital communication platforms in reaching this demographic.

Postcampaign demographics were comparable to those observed precampaign, with a similar average age of 23.3 (SD 3.0) and a predominantly female sample, consistent with the precampaign cohort (176/267, 65.9%; Table 1). Racial diversity was similar to that of the precampaign sample, and both samples included fewer than 10% Hispanic respondents. As in the precampaign group, more than half of participants were enrolled in a health professions program (146/267, 54.7%).

In the postcampaign sample only, athletic facility usage and skin type distribution were measured. Athletic facility usage was common, with 89.1% (238/267) reportedly visiting to some extent, as defined by selecting any response other than “never” on the 5-point Likert scale. Of these participants, more than half reported visiting very frequently, as indicated by selecting “often” (n=98) and “always” (n=32). This finding supported the strategic placement of additional sunscreen dispensers at athletic facilities across campus.

Skin type distribution in the postsurvey sample showed that 61.4% (164/267) identified as types I-II (lighter skin types) and 38.6% (103/267) as types III-VI (darker skin types). Skin type demonstrated clear variation across racial groups: among White participants (n=121), nearly all reported lighter skin tones, with 49.6% (60/121) identifying as type I; 38.0% (46/121) as type II; 12.4% (15/121) as type III; and none identifying above type III. Asian American or Pacific Islander participants (n=80) showed a broader distribution, including types II (36/80, 45.0%), III (24/80, 30.0%), and IV-V (17/80, 15.3%). The majority of Black or African American participants (n=17) reported darker skin tones, with 52.9% (9/17) identifying as type V and 23.5% (4/17) as type VI. Middle Eastern participants (n=22) predominantly reported intermediate tones (type II: 5/22, 22.7%; type III: 11/22, 50.0%). These distributions align with established patterns between race and skin phototype and support the validity of the self-reported classification within this cohort.

### SSC Instagram Engagement (Instagram Insights)

Engagement metrics are summarized in Table 2. Among educational content, 24 out of 43 (55.8%) posts integrated multiple themes within a single post. Sunscreen use was the most frequently represented topic, appearing in 69.8% (30/43) of all educational posts. Educational content averaged 262.2 (493.6) views and 7 (13.3) likes per-post, compared with 214.1 (270.2) views and 5.4 (7.7) likes for outreach content. On average, 14.5% (26.5%) of viewers of educational posts were nonfollowers, indicating that each educational post reached approximately 38 new users beyond the account’s existing audience. The highest performing post was a reel showing photos of an on-campus sunscreen dispenser, personified by a facial filter with isolated eyes and lips. This was subsequently followed by photos of sun-damaged skin on the face and neck overlaying popular audio saying, “Nope, I’m good, thank you,” to encourage sunscreen use. This reel generated 2947 views (nonfollowers: 2720/2947, 92%) and received n=66 likes, substantially surpassing other engagement methods.

**Table 2.** Social media engagement metrics by type.

Post type or theme	Post, n	Views, mean (SD)	Likes, mean (SD)	Nonfollowers (%), mean (SD)
Educational^a^	43	262.2 (493.6)	7.0 (13.3)	14.5 (26.5)
Wearing sunscreen	30	308.1 (586.9)	8.5 (15.4)	18.5 (30.8)
Protective clothing	16	98.3 (52.6)	1.6 (1.6)	3.6 (7.5)
Sun safety in winter	16	155.1 (155.7)	4.9 (9.6)	14.2 (26.4)
Tanning beds	12	149.1 (122.6)	2.8 (3.9)	4.8 (8.2)
Skin cancer by skin type	6	172.2 (133.1)	4.7 (5.5)	6.2 (10.2)
Outreach	19	214.1 (270.2)	5.4 (7.7)	9.9 (21.0)
Total	62	279.5 (226.0)	7.9 (6.2)	15.5 (17.1)

a Educational posts do not sum to n=43 due to the coding of multiple themes.

### SSC Instagram Awareness

At baseline, 24.1% (55/230) of precampaign participants reported awareness of the university’s SSC initiative. After the campaign, postsurvey data revealed that 48.7% (130/267) of participants reported awareness of the SSC Instagram account, demonstrating a significant increase in visibility (*χ*^2^_1_=32.5; *P*<.001). Among those who were aware, the majority (113/130, 86.9%) indicated that they followed the account. SSC Instagram awareness varied significantly by skin type: 56.7% (93/164) of participants with skin types I-II were aware of the SSC Instagram compared with 35.9% (37/103) of participants with types III-VI (*χ*²_1_=10.9; *P<*.001). Participants who followed the SSC Instagram account were also substantially more likely to report sunscreen dispenser use (*χ*²_1_=85.2; *P<*.001); the majority of followers (102/113, 90.3%) had used a dispenser.

### Content Recall

Among those who indicated aided recall of specific themes from the SSC Instagram account (n=124), 539 total recall selections were recorded across 5 educational themes. Sunscreen use was the most frequently recalled theme (121/124, 97.6%), followed by protective clothing (111/124, 89.5%), tanning bed risks (106/124, 85.5%), skin cancer by skin tone (104/124, 83.9%), and winter sun safety (97/124, 78.2%). Sunscreen was also the most frequently posted theme (30/43, 69.7% of educational posts), followed by protective clothing (16/43, 37.2% of educational posts). Notably, recall patterns did not strictly mirror posting frequency. For example, tanning bed risks appeared in only 27.9% (12/43) of education-focused posts but were recalled by 85.5% (106/124) of participants, while skin cancer by skin tone appeared in 14% (6/43) of educational posts yet achieved 83.9% (104/124) recall. These results suggest that tanning bed risks and skin cancer by skin tone may have been especially salient themes among respondents who were aware of the SSC Instagram account. However, because recall was assessed using an aided theme list and only among respondents aware of the account, these findings should be interpreted as indicators of theme salience rather than as direct evidence of knowledge gain. Winter sun safety was the third most frequently posted educational theme (16/43, 37.2% of educational posts) and was less frequently recalled (97/124, 78.2%), suggesting limited resonance.

### Sun Safety Behaviors by SSC Instagram Following

There were no demographic differences between those who did and did not follow the SSC Instagram account, with the exception of skin tone: followers were more likely to report lighter (types I-II) skin tones (*χ*²_1_=10.9; *P*<.001). Respondents who reported following the social media account (113/267, 42.3%) also reported significantly higher sun safety behaviors compared to nonfollowers (Table 3). SSC Instagram followers reported greater sunscreen use (mean 4.1, SD 0.8 vs mean 3.3, SD 1.1; *P<*.001), more protective clothing use (mean 3.5, SD 0.8 vs mean 3.2, SD 1.0; *P*=.007), increased shade-seeking behaviors (mean 3.8, SD 0.8 vs mean 3.1, SD 1.1; *P<*.001), and more frequent UV index checking (mean 2.8, SD 0.8 vs mean 2.2, SD 1.2; *P*<.001). The composite sun safety behavior score was also significantly higher among followers than nonfollowers (mean 24.9, SD 4.5 vs mean 18.5, SD 4.7; *P<*.001).

**Table 3.** Sun safety behaviors by Skin Smart Campus (SSC) Instagram following status among postsurvey respondents (n=267).

Behavior	Instagram followers (n=113), mean (SD)	Nonfollowers (n=154), mean (SD)	*t* test (*df*)	*P* value
Sunscreen use	4.1 (0.8)	3.3 (1.1)	6.02 (265)	<.001
Protective clothing	3.5 (0.8)	3.2 (1.0)	2.73 (265)	.007
Shade seeking	3.8 (0.8)	3.2 (1.0)	5.53 (265)	<.001
UV Index checking	2.8 (0.8)	2.2 (1.2)	5.21 (265)	<.001
Total	24.9 (4.5)	18.5 (4.7)	11.12 (265)	<.001

In a multivariable logistic regression analysis adjusting for skin tone (odds ratio [OR] 2.33, 95% CI 1.19‐4.56; *P<*.001) and sunscreen dispenser use (OR 7.78, 95% CI 3.82‐15.84; *P<*.001), following the SSC Instagram account remained a strong independent factor associated with higher postcampaign sun safety behavior (Table 4). Participants who checked or followed the SSC Instagram account were over 6 times more likely to have a higher sun safety behavior composite score compared to nonfollowers (OR 6.81, 95% CI 3.41‐13.60; *P<*.001). This finding should be interpreted as an association rather than evidence of causal impact, as participants who were already more interested in sun safety may have been more likely to follow the SSC Instagram account.

**Table 4.** Multivariable logistic regression of sun safety behavior at posttest (N=267)^a^.

Factor	OR^b^ (95% CI)	*P* value
Skin tone (lighter vs darker^c^)	2.33 (1.19‐4.56)	.01
Sunscreen dispenser use (yes vs no^c^)	7.78 (3.82‐15.84)	<.001
SSC^d^ Instagram following (yes vs no^c^)	6.81 (3.41‐13.60)	<.001

a Sun safety behavior composite scores range from 7‐35. High sun safety behavior is defined as a composite score >median value (22).

b OR: odds ratio.

c Model referent.

d SSC: Skin Smart Campus.

## Discussion

This study evaluated a social media campaign implemented as part of a multicomponent sun safety initiative on a university campus, with a focus on how group-targeted and tailored digital communication shaped engagement, awareness, message recall, and early behavioral indicators among young adults. The broader implementation outcomes of the SSC initiative, including pre-post changes in knowledge, dispenser use, and overall sun safety behavior, were reported by Perez et al [41]. The analysis extends that work by focusing on the Instagram campaign’s reach, engagement, aided thematic recall, and associations between Instagram following and sun safety behaviors. This anonymous pre-post analysis concentrated on how message design, theme selection, and platform-specific strategies influenced campaign reach and resonance within the young adult population.

### Principal Findings

Consistent with prior work demonstrating the central role of social media as a source of health information among young adults [2,3,36], the SSC Instagram achieved substantial visibility. Trend-based posts helped embed sun protection behaviors within campus culture, increase perceived social norms around sunscreen use, and foster peer-to-peer diffusion of knowledge through humor and familiarity with current digital trends. This creative approach aimed to normalize daily sunscreen use and UVR protective practices by aligning them with the aesthetic and cultural norms of the digital environment. Rather than viewing sun safety in isolation, the campaign leveraged young adults’ focus on appearance—such as by highlighting the aesthetic impact of sun damage—to reframe protective practices as culturally-relevant health behaviors.

The peer network dissemination strategy adequately leveraged trusted peer groups to boost message reach among young adults unlikely to encounter institutional channels. Campus organizations that reposted SSC Instagram content had a potential digital reach equivalent to roughly one-third of the student body. Additionally, Instagram analytics revealed that educational posts routinely reached Instagram users who did not follow the SSC account, suggesting that peer network dissemination effectively expanded exposure beyond the immediate audience. This pattern is notable given that campaigns relying solely on organic reach often struggle to penetrate beyond existing followers [38,39].

At the platform level, exposure among Instagram users who did not follow the SSC account remained a meaningful contributor to reach, with 14.5% (38/262.2) of total impressions generated by users not subscribed to the account, on average. Shares and comments were infrequent, consistent with prior young adult-focused health promotion research [38,39]. However, views and likes demonstrated consistent performance across themes. Together, these analytics indicate that the SSC Instagram campaign achieved broad algorithmic circulation and engagement.

The high performance of sunscreen-focused reels, especially those incorporating humor and trend-based formats, highlights the importance of aligning prevention messaging with the aesthetic and cultural norms of the young adult digital environment. This environment is uniquely characterized by identity exploration in public spaces, high social visibility and constant comparison, and blended online and offline personas. Moreover, it has been described as an algorithm-shaped experience, with participatory creativity and interactivity, and one that is influenced by rapid cultural cycles and community-seeking across real and digital borders [51]. Young adult health engagement on social media is also often shaped by influencer-driven norms and intuitive assessments of credibility rather than formal expertise [2]. These dynamics can amplify both positive and negative health influences, emphasizing the need for group-tailored, trend-aligned, and inclusive messaging. The SSC Instagram attempted to leverage these characteristics to increase campaign reach and appears to have successfully done so within a university campus environment.

Content recall patterns provide insight into the salience of SSC Instagram themes among respondents who were aware of the account. Because recall was assessed using a list, these findings should be interpreted as aided thematic recall rather than unaided memory for specific posts. Although sunscreen was the most frequently posted topic, high levels of aided recall were also observed for tanning bed risks and skin cancer by skin tone, which were posted less often but may have had strong salience for this audience. These findings suggest that message novelty, perceived personal relevance, and the counter-messaging nature of content (eg, correcting misconceptions about risk across skin tones) may exert greater influence on recall than posting frequency alone. This pattern may also reflect the comparatively low baseline knowledge observed for tanning bed risks and skin cancer susceptibility across skin tones in the precampaign survey, suggesting that participants may have been more likely to notice and remember content that addressed these existing knowledge gaps. Importantly, pre-post knowledge outcomes for the broader SSC initiative are reported elsewhere [41]; the present findings focus on social media–specific recall and engagement only. While direct comparative evidence evaluating message characteristics versus posting frequency on recall is limited, prior literature demonstrates that message tailoring, narrative framing, and counter-messaging can improve salience and resonance [2,3,27,52-54].

The low recall of winter sun safety content, despite being one of the more frequently posted themes, underscores limitations in seasonal messaging for UVR prevention. Young adults may underestimate winter UVR exposure, which is consistent with previous literature showing lower perceived risk during colder months [55,56]. These findings suggest that future campaigns may need to intensify or diversify winter messaging modalities (eg, in-person prompts, location-based mobile reminders) to overcome entrenched assumptions about seasonal risk.

Patterns of campaign awareness also reveal important disparities. Young adults with lighter skin tones were significantly more likely to be aware of the SSC Instagram account, consistent with evidence that individuals with darker skin tones often perceive themselves to be at lower risk for skin cancer and are less likely to seek UVR-related health information [57,58]. This gap has substantive equity implications: without tailored efforts to engage young adults across diverse skin tones, digital prevention strategies may inadvertently reinforce disparities in UVR protection behaviors. The strong recall of the “skin cancer by skin tone” theme suggests that inclusive, identity-relevant messaging is not only feasible but impactful and should be expanded in future iterations of the campaign.

The integration of digital messaging with environmental supports also appeared meaningful, specifically with the installation of seven sunscreen dispensers. Young adults who followed the SSC Instagram account and those who visited athletic centers were more likely to use campus sunscreen dispensers, illustrating how digital and built environments can mutually reinforce one another. Similar synergistic effects have been observed in other public health interventions, such as campus-based human papillomavirus or influenza vaccination campaigns that paired social media outreach with on-site clinics, resulting in increased vaccine uptake [59,60]. Further research is warranted to elucidate the value of integrating digital and physical components to maximize healthy behavior change. Still, this multicomponent approach aligns with ecological models of behavior change, which emphasize the interaction of personal, social, and environmental factors in shaping preventive health behaviors [61].

Several implications emerge from this evaluation. First, social media campaigns targeting young adults benefit substantially from high-quality tailoring and trend-aligned design, which increase relevance and engagement. Second, theme selection should not rely solely on frequency; topics that address misconceptions, highlight disparities, or provide counter-narratives may yield disproportionately strong recall. Third, equitable campaign impact requires intentional efforts to reach populations with historically lower UVR-related risk perception, including young adults with darker skin tones. Finally, pairing digital campaigns with accessible environmental resources, such as on-campus sunscreen dispensers, may enhance both awareness and behavioral uptake.

### Limitations

This study has several limitations that should be kept in mind when interpreting the findings. The anonymous, independent-samples design prevents linking participants across time and limits inferences about within-person change. Moreover, participant overlap cannot be determined. The nonexperimental design further constrains causal interpretation because exposure was not manipulated and participants were not randomly assigned to conditions; accordingly, findings should be interpreted as descriptive and associative. The observed association between Instagram account engagement and sun safety behaviors may also reflect reverse causality, such that individuals already more engaged in sun protection were more likely to seek out or attend to campaign content, and may be influenced by common method variance given that both exposure-related and behavior-related variables were self-reported, which can inflate observed associations. More broadly, self-reported survey data are subject to recall and social desirability bias. In addition, Instagram analytics provide aggregate rather than individual-level metrics, limiting the precision of linking digital exposure to behavioral outcomes, and are influenced by platform algorithms and privacy protections that shape how content is distributed and which users are reached; as a result, engagement indicators such as likes and views may not fully capture passive exposure or reflect the true extent of audience reach. The study was also conducted at a single urban university, which may limit generalizability to other campus environments or institutional settings, and Fitzpatrick skin type was collected only in the postcampaign survey. Thus, it was not possible to evaluate pre-post sample comparability or change by skin type.

Nonetheless, this study contributes an important perspective to the literature on digital UVR prevention strategies by demonstrating how social media can function as a high-reach, low-cost, and culturally embedded medium for public health messaging among young adults. As preventive medicine increasingly adapts to digital platforms, these findings highlight the value of targeted, inclusive, and visually engaging communication strategies that resonate with contemporary media habits while supporting equitable access to cancer-preventive behaviors.

### Conclusions

Taken together, this social media–focused SSC evaluation illustrates the promise and ongoing challenges of delivering sun safety campaigns within the dynamic digital landscape that shapes young adult communication and identity. By aligning message design with platform norms, cultural aesthetics, and the interaction patterns that characterize young adult social media use, the SSC Instagram campaign achieved meaningful reach, strong aided thematic recall, and tangible associations with early behavioral indicators. At the same time, our findings underscore the need for continued attention to equity, seasonal risk perception, and the integration of digital and environmental supports in order to strengthen the impact of future iterations. As universities increasingly look to social platforms as core channels for health promotion, this research demonstrates how thoughtful, audience-centered design can translate public health goals into strategies that resonate within the fast-moving and socially mediated environments of young adults.

## Abbreviations

HIPAA: Health Insurance Portability and Accountability Act
OR: odds ratio
SSC: Skin Smart Campus
UVR: UV radiation
